# The effectiveness of interventions to reduce the transmission of acute respiratory infections in care homes: a systematic review

**DOI:** 10.1093/pubmed/fdae178

**Published:** 2024-08-13

**Authors:** Merlin L Willcox, Deepthi Lavu, Usaid Yousaf, Sam Dalton, Nia Roberts, Annette Plüddemann

**Affiliations:** Primary Care Research Centre, School of Primary Care, Population Sciences and Medical Education, University of Southampton, Southampton S016 5ST, UK; APEX (Exeter Collaboration for Academic Primary Care), Department of Health and Community Sciences, Faculty of Health and Life Sciences, University of Exeter, Exeter EX1 2LU, UK; Primary Care Research Centre, School of Primary Care, Population Sciences and Medical Education, University of Southampton, Southampton S016 5ST, UK; Primary Care Research Centre, School of Primary Care, Population Sciences and Medical Education, University of Southampton, Southampton S016 5ST, UK; Bodleian Healthcare Libraries, University of Oxford, Oxford OX1 2JD, UK; Nuffield Department of Primary Care Health Sciences, University of Oxford, Radcliffe Observatory Quarter, Woodstock Rd, Oxford OX 2 6GG, UK

**Keywords:** acute respiratory infections, antiviral prophylaxis, care homes, infection control, prevention, systematic review

## Abstract

**Background:**

Care home residents are at high risk from outbreaks of respiratory infections, such as influenza and COVID-19. We conducted a systematic review of randomized controlled trials, to determine which interventions (apart from vaccines) are effective at reducing transmission of acute respiratory illnesses (ARIs) in care homes.

**Methods:**

We searched CINAHL, Medline, Embase and Cochrane for randomized controlled trials (RCTs) of interventions to prevent transmission of ARIs in care homes (excluding vaccines), to April 2023.

**Results:**

A total of 21 articles met inclusion criteria. Two infection control interventions significantly reduced respiratory infections. Oseltamivir significantly reduced risk of symptomatic laboratory-confirmed influenza (OR 0.39, 95%CI 0.16–0.94, three trials), and influenza-like illness (OR 0.50, 95%CI 0.36–0.69), even in a vaccinated population. High dose vitamin D supplementation reduced incidence of ARIs (incidence rate ratio 0.60; 95%CI 0.38–0.94, one trial). Nine other RCTs of vitamin, mineral, probiotic and herbal supplements showed no significant effect.

**Conclusion:**

Transmission of respiratory infections in care homes can be reduced by educational interventions to improve infection control procedures and compliance by staff, by antiviral prophylaxis soon after a case of influenza has been detected, and by supplementation with high-dose Vitamin D3. Further research is needed to confirm the effect of high-dose Vitamin D3.

## How this fits in

NICE guidelines recommending antiviral prophylaxis for care home residents, during influenza outbreaks, are often not implemented. This research shows that antiviral prophylaxis can halve the risk of influenza even in vaccinated care home residents, if given within 7 days of the first case in the care home.

Current NICE guidelines recommend supplementation with Vitamin D at 400 IU daily. This research shows that high-dose supplementation (100 000 IU per month) can reduce risk of respiratory infections by 40%.

## Introduction

The UK has over 167 00 care homes with an estimated population of >440 000 residents.^1^ They are susceptible to outbreaks of infections, most commonly acute respiratory infections (ARIs),^2^^,^^3^ because of their age and comorbidities,^4^^,^^5^ which impair their immune response.^6^ Furthermore, the care home environment facilitates transmission through direct physical contact (care givers in nursing homes spend up to 40% of their time providing direct personal care to residents^7^), aerosols and fomites (in communal areas and shared facilities).^8^ Person-to-person spread is the most common route of transmission in outbreaks,^3^ and COVID-19 presented the additional challenge of asymptomatic transmission.^9^^,^^10^ Standard infection control measures and vaccines alone may not be enough to prevent infections.^8^ Over 45 000 residents died of COVID-19 during the pandemic,^11^ ~20% of all UK deaths from COVID-19. In some homes, over a quarter of residents died in a short time.^12^

This review aims to assess and compare the effectiveness (and safety) of interventions (apart from vaccines) for reducing transmission of ARIs in care homes.

## Methods

The systematic review protocol is registered on Prospero (CRD42021292849).

### Search strategy

We searched MEDLINE(OvidSP)[1946-], Embase(OvidSP)[1974-], EuropePMC,^13^ CENTRAL and CDSR^14^ and ClinicalTrials.gov,^15^ to April 2023. We searched using subject headings and author keywords for our main concepts of care homes and respiratory infections (Supplementary Table 1). Methodological filters were applied to limit to RCTs.^16^^,^^17^ No date or language limits were applied. Results were exported to Endnote 20 for deduplication.

### Study selection

Two reviewers independently screened titles, abstracts and full texts against the inclusion/exclusion criteria in Rayyan.^18^ Disagreements were resolved through discussion with a third reviewer. We included only RCTs of interventions designed to reduce transmission of ARIs in care home residents, compared with placebo, usual care or another intervention, reporting at least one relevant outcome in care home residents: incidence of ARIs, hospitalization and mortality from ARIs, occurrence of outbreaks, and incidence of side-effects. We excluded studies on vaccines, aspiration pneumonia and artificial life support.

### Data extraction and synthesis

Two reviewers independently used the Cochrane Risk of Bias tool 2^19^ to assess quality, and disagreements were resolved by discussion with a third reviewer. Two reviewers extracted data on characteristics of included studies and on relevant outcomes. Where two or more studies reported the same outcome for the same intervention, we planned to meta-analyse these using RevMan.^20^ For studies reporting odds ratios or absolute figures, we used the dichotomous data tool with the ‘Mantel–Haenszel’ method and a random effects analysis.

## Results

### Included studies

**Fig. 1:**
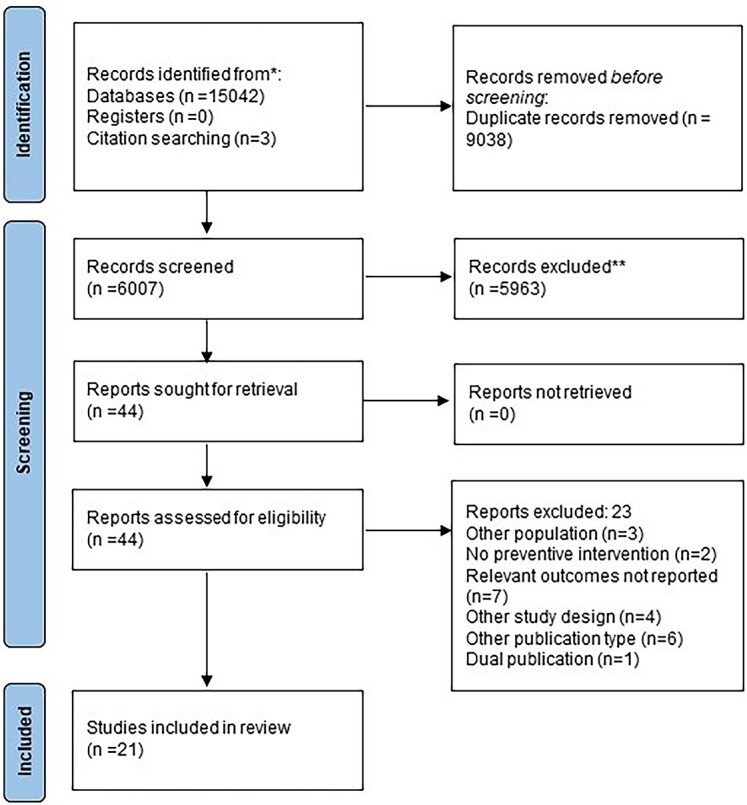
PRISMA flowchart.

The literature search found 6007 articles after removing duplicates (Fig. 1). Twenty-one articles met our inclusion criteria.

### Study characteristics

Four cluster RCTs assessed the impact of behaviour-change interventions on care home staff infection control practices,^21–24^ mostly focussing on hand hygiene, through education, reminders and provision of alcohol hand rubs (Table 1a).

**Table 1:** Characteristics of included studies

a: Cluster RCTs of interventions to improve infection control practices of care home staff										
Reference	Country	Setting	Number of participants	Vaccination status	Time of study	Intervention	Comparison	Relevant outcome(s)	Duration of follow-up	Risk of bias
Makris et al, 2007	USA	8 LTCFs in New Jersey and Delaware	890	Not reported	not reported	Infection control educational program	Existing infection control policies and procedures	Total infection rate and % change in ARI rate	NS	High
Ho et al, 2012	Hong Kong	18 residential homes	2407	Not reported	November 2009 to July 2010	Alcohol based hand rubs, gloves, talks on hand hygiene	Health talks about topics other than hand hygiene (eg healthy eating, environmental hygiene, exercise)	Outbreaks of respiratory infections before and after intervention	7 months	High
Teesing et al, 2021	Netherlands	66 nursing homes	Not reported	Not reported	Oct 2016 - Oct 2017	Hand hygiene e-learning, live talks, audit	No intervention	Incidence of influenza-like illness (ILI), assumed pneumonia	10 months	High
Yeung et al, 2011	Hong Kong	6 nursing homes	675	Not reported	April–October 2007	2-hour seminar on hand hygiene, alcohol hand rub	Training on Basic life support program	Incidence of pneumonia, outbreaks of influenza	7 months	Some concerns

(*Continued*)

**Table 1:** Continued

b: RCTs of antiviral prophylaxis for residents ± staff during outbreaks of viral respiratory infections																
Reference	Study Design	Country	Setting	Participants	Number of participants	% vaccinated against influenza	Time of study	Timing for intervention	Antiviral prophylaxis	Dose	Duration (days)	Comparison	Treatment of infected patients (both groups)	Relevant outcome(s)	Follow-up (days)	Risk of Bias
Ambrozaitis et al, 2005	RCT	Lithuania, Netherlands, and Israel	12 nursing homes	Residents, able to inhale from a Diskhaler	489	9%	3 influenza seasons (1997–2000)	Influenza outbreak declared^1^	Zanamivir	10 mg inhaled od	14	Placebo inhaler	NS	Symptomatic laboratory confirmed influenza	15	Some concerns
Gravenstein et al, 2005	RCT	USA	9 nursing homes in rural Wisconsin	Nursing home residents	375	98%	November 1997 to March 2000	Influenza outbreak declared^2^	Zanamivir	10 mg inhaled od	14	Influenza A: rimantadine 100 mg od for 14d. Influenza B: Placebo	NS	Symptomatic laboratory confirmed influenza	14	Low
Schilling et al, 1998	cRCT	USA	1 nursing home in rural Wisconsin	Nursing home residents	257	Not reported	Nov 1996 - April 1997	Influenza outbreak declared^2^	Zanamivir	10 mg inhaled twice daily +4.4 mg intranasal twice daily	14	Influenza A: rimantadine 100 mg od for 14d. Influenza B: Placebo	NS	Incidence of laboratory-confirmed influenza	14	High
Booy et al, 2012	cRCT	Australia	16 Residential homes	Staff and residents	not reported	83–85%	30 June 2006–23 Dec 2008	Influenza outbreak declared^3^	Oseltamivir	75 mg od	10	No prophylaxis	75 mg oseltamivir twice daily for 5 days	Attack rate of influenza or deaths, hospitalization, pneumonia, and adverse drug reactions.	Duration of outbreak (up to 26d)	High
Peters et al, 2001	RCT	USA, France, Netherlands, Belgium, UK	31 Residential homes and sheltered accommodation	Frail older occupants of care homes.	548	80%	1998–1999 influenza season	Influenza outbreak^4^	Oseltamivir	75 mg once daily	42	Placebo	NS	Laboratory confirmed clinical influenza	42	Low
Van der Sande at al, 2014	cRCT	Netherlands	42 Nursing homes.	Frail elderly nursing home residents.	140	Oseltamivir group: 100%; placebo group: 81%	2009–2013	Influenza outbreak^5^	Oseltamivir	75 mg once daily	10	Placebo	oseltamivir 75 mg twice daily for 5 days	Laboratory confirmed clinical influenza or a clinical diagnosis of ILI	10	High
Cohen et al, 2021	RCT	USA	74 Nursing homes and assisted living facilities	Staff and residents at nursing homes.	1175	NA	2 August to 20 November 20 2020	Within 7 days of a reported confirmed SARS-CoV-two case	Bamlanivimab	4200 mg, single intravenous infusion	1	Placebo	Bamlanivimab	Incidence of COVID 19	56	Low

(*Continued*)

**Table 1:** Continued

c: RCTs of nutritional supplements given to care home residents													
Reference	Country	Setting	Population	Number of participants	Vaccination status	Time of study	Intervention	Dose	Duration	Comparison	Relevant outcome(s)	Duration of follow-up for primary outcome	Risk of Bias
Liu et al, 2007	Canada	21 nursing homes around Toronto	Nursing home residents	763	Influenza vaccine: 77–80%; pneumococcal vaccine: 70–73%	not reported	Multivitamin and mineral supplement	One tablet daily	19 months	placebo	Incidence of respiratory tract infections	18 months	Low
Meydani et al, 2004	USA	33 long-term care facilities in the Boston, Massachusetts area	Nursing home residents	617	Influenza vaccine: 100%; Pneumococcal vaccine: 13% of intervention group, 9% of placebo group	April 1998 to August 2001	Vit E + multivitamin and mineral capsule	200 IU daily	Continuous	placebo (contained 4 IU of Vitamin E) + multivitamin and mineral capsule	Incidence of respiratory infections	317–321 days	Some Concerns
Ginde et al, 2017	USA	25 Colorado nursing and residential homes	Older residents (≥60 years) of long-term care facilities	107	Not reported	June 2010 to January 2014	Vitamin D3 - high dose supplement	100,000 IU monthly	Continuous	standard dose Vitamin D (400–1000 IU daily)	Incidence of ARI	12 m	Low
Godan Hauptman et al, 2021	Croatia	2 nursing homes in Zagreb County	Nursing home residents, all deficient in Vitamin D	97	Influenza vaccine: 100%	October 2016 to August 2017	Vitamin D3 -standard dose supplement	800 IU daily	3 months, starting on day of vaccination	no Vitamin D supplementation	Incidence of influenza (confirmed)	3 m	High
Girodon et al, 1999	France	25 nursing homes	Nursing home residents	725	Not reported	April 1992–April 1993	Zinc + selenium OR Vitamins A + C + E OR both	Zn 20 mg; Se 100mcg; vit A 6 mg; vit C 120 mg; Vit E15mg	Continuous	placebo	Occurrence of ARI	24 m	Some concerns
Butler et al, 2020	UK	23 residential and nursing homes	Care home residents, aged 65 years and older; 50% were severely frail	310	Not reported	Dec 2016 - May 2018	*Probiotic: Lactobacillus rhamnosus* GG + *Bifidobacterium animalis* subsp lactis BB-12	One capsule daily	Continuous	Placebo	Incidence of respiratory tract infections; adverse events	12 months	Low
Van Puyenbroeck et al, 2021	Belgium	53 Nursing homes in Antwerp	Healthy nursing home residents aged ≥65 years	737	Influenza vaccine: 100%, given 3 weeks after starting probiotic	Oct 2007 - April 2008	Probiotic: *Lactobacillus casei* Shirota (LcS)	One bottle bd of fermented milk containing ≥6.5 x 10^9^ live LcS/bottle	6 months	Placebo	Number of days with respiratory symptoms, the probability of respiratory symptoms	6 m	Some concerns
Fonolla et al, 2017	Spain	5 nursing homes in Granada	Nursing home residents, > 65 years of age	98	Influenza vaccine: 100%, given 2 weeks after starting probiotic	Oct 2015 - April 2016	Probiotic: *Lactobacillus coryniformis* K8 CECT5711	One capsule containing 3 × 10^9^ CFU/day	14 days	Placebo (300 mg maltodextrin)	Incidence of influenza-like illness (ILI)	6 m	High
Gracian-Alcaide et al, 2020	Spain	One nursing home in Granada	Nursing home residents > 65 years of age	65	Influenza vaccination: 100%	Dec 2019 - March 2020	Elderberry (*Sambucus nigra* L.) dried fruit juice + Reishi (*Ganoderma lucidum*) aqueous extract	1.5 g Elderberry +0.5 g Reishi daily	14 weeks	placebo	incidence of respiratory tract infections, adverse events	14 weeks	Some concerns
Wong et al, 2012	Hong Kong	10 elderly centres and old age homes	Residents aged 60 or above	740	Not reported	Dec 2003 to July 2004	Sachet of granules containing 12 Chinese herbs*	4 g sachet of herbal preparation on alternate days	8 weeks	Placebo	Number of influenza episodes and influenza-like illnesses detected.	12 weeks	Some concerns

***Definitions of influenza outbreak:***

1: at least one confirmed case of influenza by viral culture, and at least 10 residents or 10% with new respiratory symptoms

2: at least one confirmed case of influenza in last 7 days, and at least 10% with new respiratory symptoms

3: at least one confirmed case of influenza, and at least two residents with ILI over 3 days, or 3 over 7 days

4: at least one confirmed case of influenza in the residential home OR at least 2 confirmed cases of influenza in the immediate geographic community within the previous 7 days

5: at least one confirmed case of influenza in residents with ILI

Seven trials (three cluster, four individually randomized) evaluated the use of prophylactic antivirals given to residents during ARI outbreaks, when at least one other resident had virological confirmation of influenza (six trials) or COVID-19 (one trial, Table 1b).^25–31^

The remaining 10 RCTs studied preventative administration of nutritional supplements (Table 1c): Vitamin D3 (two trials),^32^^,^^33^ Vitamin E (one trial),^34^ multivitamin/mineral supplements (two trials),^35^^,^^36^ probiotics (three trials),^37–39^ and herbals (two trials).^40^^,^^41^

### Risk of bias

Most cluster-randomized trials of infection control or antiviral prophylaxis were at high risk of bias (ROB) (Table 2a). These interventions were delivered via care home staff, who could not be blinded. Staff were responsible for reporting infections, and those in the intervention group may have been more aware and more likely to report.^23^ Although adherence to hand hygiene improved, levels were low even in intervention facilities. Some trials also reported inadequate adherence to antiviral prophylaxis, which was not accounted for in the analysis.

**Table 2:** Risk of bias of included studies

a: Cluster RCTs of infection control/antiviral prophylaxis								
Reference	Cochrane Risk of Bias 2 domain							Overall
	1a	1b	2a	2b	3	4	5	
Trials of infection control interventions								
Ho et al, 2012	L	L	L	SC	L	L	H	H
Makris et al, 2000	L	L	L	SC	SC	H	SC	H
Teesing et al, 2021	L	L	SC	H	L	H	L	H
Yeung et al, 2011	L	L	L	SC	L	L	SC	SC
Trials of antiviral prophylaxis								
Booy et al, 2012	L	L	H	H	L	H	SC	H
Schilling et al, 1998	SC	L	SC	SC	L	H	SC	H
Van Der Sande et al, 2014	SC	L	H	H	L	L	H	H
*b: Individually randomised controlled trials of antiviral prophylaxis or nutritional supplements*								
*Reference*	*Cochrane ROB2 Domain*						*Overall*	
	*1*	*2a*	*2b*	*3*	*4*	*5*		
Trials of antiviral prophylaxis								
Ambrozaitis et al, 2005	L	L	L	L	L	SC	SC	
Cohen et al, 2021	L	L	L	L	L	L	L	
Gravenstein et al, 2005	L	L	L	L	L	SC	SC	
Peters et al, 2001	L	L	L	L	L	SC	SC	
Trials of preventative herbal supplements								
Gracian-Alcaide et al, 2020	L	L	L	L	L	SC	SC	
Wong et al, 2013	L	L	L	L	L	SC	SC	
Trials of probiotic supplements								
Butler et al, 2020	L	L	L	L	L	L	L	
Fonolla et al, 2019	L	H	L	SC	L	L	H	
Van Puyenbroeck et al, 2012	L	L	H	L	L	SC	H	
Trials of vitamin/mineral supplements								
Ginde et al, 2017	L	L	L	L	L	L	L	
Girodon et al, 1999	L	L	L	L	L	SC	SC	
Godan Hauptman et al, 2021	SC	H	H	L	SC	SC	H	
Liu et al, 2007	L	L	L	L	L	SC	SC	
Meydani et al, 2004	L	L	L	L	L	SC	SC	

Key:

L = Low; SC = Some concerns; H = High

1a: Risk of bias arising from the randomization process

1b: Risk of bias arising from the timing of identification or recruitment of participants

2a: Risk of bias due to deviations from the intended interventions (effect of assignment to intervention)

2b: Risk of bias due to deviations from the intended interventions (effect of adhering to intervention)

3: Risk of bias due to missing outcome data

4: Risk of bias in measurement of the outcome

5: Risk of bias in selection of the reported result

Most individually randomized trials had lower ROB, chiefly because it is easier to conduct a double-blind trial using identical placebos for individual controls. However, only two trials reported following a pre-specified statistical analysis plan (Table 2b). Five of the seven trials of antiviral prophylaxis were industry-funded.^25^^,^^27–30^

### Infection control interventions

Infection control interventions had varying impacts on hand hygiene behaviours by care home staff. The best improvements were achieved in Hong Kong,^21^ where hand hygiene compliance improved from 27.0% (at baseline) to 60.6% (after 4 months) with the intervention, compared with no significant change in the control group (*P* < 0.001). Another study in Hong Kong^24^ showed that use of alcohol hand rubs increased from 1.5% to 15.9%, but hand washing reduced in the intervention group. In the Netherlands,^23^ adherence in the intervention group increased from 12% to 36%, significantly better than the control arm (OR: 2.28; 95%CI:1.67–3.11). The fourth study did not measure adherence.^22^

Three studies reported incidence of pneumonia. These could not be combined in meta-analysis because outcomes were not directly comparable. In the USA,^22^ incidence of lower respiratory tract infections (LRTIs) in intervention homes decreased non-significantly, compared to control homes (median 24.8% and 13.6% decrease, *P* = 0.19). In the Netherlands^23^ incidence of pneumonia also decreased non-significantly (adjusted incidence rate ratio (IRR) 0.87, 95%CI 0.60–1.26, *P* = 0.47). Incidence of pneumonia requiring hospitalization reduced significantly in one Hong Kong trial^24^ (IRR 0.52, 95%CI 0.29–0.96, *P* = 0.03), but there were no influenza outbreaks at all in the intervention or control groups.^24^ Risk of respiratory outbreaks was significantly reduced in intervention homes in the second Hong Kong study (IRR 0.12, 95%CI 0.01–0.93, *P* = 0.04).^21^

Incidence of Influenza-Like-Illness (ILI) was only reported in one study, which showed a significant reduction in intervention homes compared to control homes (IRR 0.51, 95%CI 0.31–0.82).^23^ A non-significant reduction in upper respiratory tract infections (URTIs) was reported in the USA^22^ (median reduction 58.4% in intervention homes, 33.1% in control homes, *P* = 0.06).

### Antiviral prophylaxis

All three studies on oseltamivir versus placebo for prophylaxis during a confirmed outbreak reported incidence of symptomatic laboratory-confirmed influenza (SLCI) as the primary outcome, although the precise definition of ‘symptomatic’ varied. All three studies required cough or another respiratory symptom, two studies also required a recorded temperature of ≥38°C or ≥ 37.2°C,^26^^,^^29^ and two required at least one systemic symptom.^29^^,^^31^ The duration of prophylaxis was 10 days in two trials^26^^,^^31^ and 6 weeks in the third,^29^ which reported a larger effect. Meta-analysis showed a significant reduction in the odds of SLCI (OR 0.39, 95%CI 0.16–0.94, Fig. 2.1). Two trials reported impact on all ILI (not only laboratory-confirmed), which showed a similar reduction (OR 0.50, 95%CI 0.36–0.69, Fig. 2.2). Two trials also reported all cases of laboratory-confirmed influenza, including those which did not meet criteria for ‘symptomatic’.^29^^,^^31^ These trials showed a smaller reduction, not statistically significant (OR 0.59, 95% CI 0.32–1.08, Fig. 2.3).

**Fig. 2:**
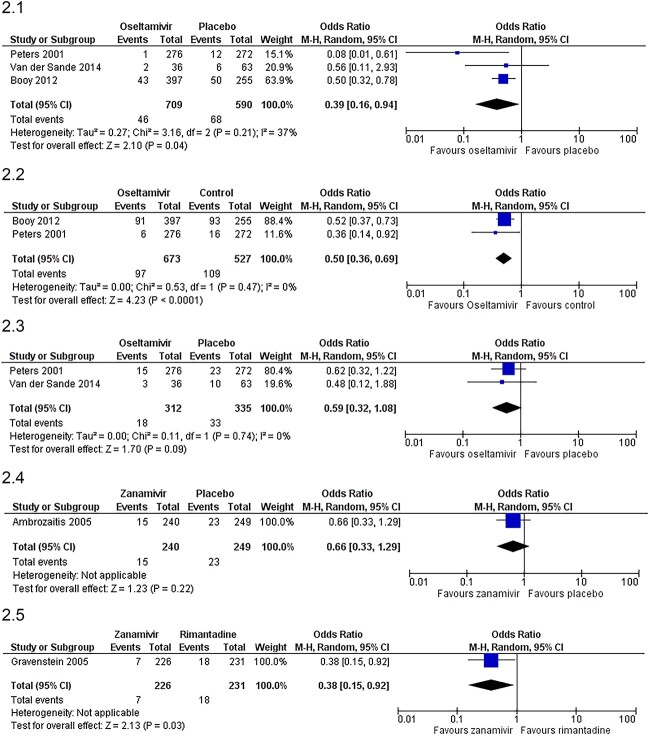
Effect of antiviral prophylaxis on incidence of influenza, (2.1) Effect of oseltamivir prophylaxis on risk of symptomatic, laboratory-confirmed influenza (SLCI). (2.2) Effect of oseltamivir prophylaxis on risk of Influenza-Like Illness (including both laboratory-confirmed and not confirmed). (2.3) Effect of oseltamivir prophylaxis on risk of all Laboratory-Confirmed Influenza (includes cases which did not meet the criteria for ‘symptomatic’). (2.4) Effect of zanamivir prophylaxis versus placebo on risk of Symptomatic, Laboratory-Confirmed Influenza (SLCI). (2.5) Effect of zanamivir prophylaxis versus rimantadine on risk of Symptomatic, Laboratory-Confirmed Influenza (SLCI).

Results from the three zanamivir studies could not be combined because they had different comparators and outcomes. Compared with placebo, zanamivir prophylaxis reduced risk of SCLI but this was not statistically significant (OR 0.66, 95%CI 0.33–1.29, Fig. 2.4).^25^ Compared with rimantadine, zanamivir significantly reduced risk of SCLI (OR 0.38, 95%CI 0.15–0.92, Fig. 2.5).^28^ The third study was a small pilot trial which reported no cases of SCLI in either of the group, and only one case of asymptomatic laboratory-confirmed influenza in the rimantadine group.^30^

One trial showed a significant effect of a single dose of intravenous bamlanivimab versus placebo (given within 7 days of a confirmed case of COVID-19) on the incidence of COVID-19 in the subsequent 8 weeks^27^ (OR 0.20, 95%CI 0.08–0.49).

Reported incidence of adverse events varied widely between trials, mainly due to duration of follow-up, but there were no significant differences between treatment and control groups in any of the studies. In the trial which gave oseltamivir for 6 weeks, 60% reported adverse events, in both treatment and placebo groups.^29^ In contrast, only 5.6% of participants on oseltamivir for 10 days reported adverse events (compared with 7.9% on placebo). With inhaled zanamivir, 32.3% reported adverse events, compared with 36.5% on placebo.^25^ With bamlanivimab, 20.1% of participants reported adverse events, compared with 18.9% in the placebo group.^27^

### Nutritional supplements

#### Vitamins

Two trials of vitamin D3 tested different doses and produced contrasting results. High-dose vitamin D3 (100 000 IU monthly) significantly reduced incidence of ARIs compared to standard dose (400–1000 IU daily) (IRR 0.60; 95%CI 0.38–0.94; *P* = 0.02, low ROB).^32^ In contrast, in a trial at high ROB, standard dose vitamin D3 (800 IU daily) had no effect compared with no treatment and indeed was insufficient to correct the deficiency which was present in most participants.^33^

Vitamin E supplementation had no significant impact on incidence of respiratory infections (IRR 0.91, 95%CI 0.77–1.06).^34^ Two trials of long-term daily multivitamin and mineral supplements found no effect on risk of respiratory infections (IRR 0.91, 95%CI 0.80–1.05^36^; OR for one or more infections, vitamin + mineral compared with placebo, 1.27, 95%CI 0.84–1.93^35^).

#### Probiotics

Three trials assessed daily probiotic supplementation (*Lactobacillus* species), but none showed a statistically significant effect. One trial found no impact of daily probiotics for one year on incidence of upper respiratory infections (adjusted IRR 1.13, 95%CI 0.71–1.78) or lower respiratory infections (adjusted IRR 1.4, 95%CI 1.1–1.9).^37^ Another trial gave probiotics for 6 months, starting 3 weeks before influenza vaccination,^39^ but found no effect on number of days with respiratory symptoms (*P* = 0.34) or number of participants with respiratory symptoms (*P* = 0.33). There was a non-significant reduction in the risk of severe respiratory tract infection (RTI) (OR 0.59, 95%CI 0.34–1.05). The third trial gave probiotics for only 14 days, immediately before influenza vaccination.^38^ Improved seroconversion to the vaccine was reported, and a non-significant reduction in incidence of ILI over 6 months (IRR 0.54, *P* = 0.19).

#### Herbal supplements

Two studies examined the impact of daily herbal supplements –a complex Chinese formula containing 12 herbs for 8 weeks,^41^ and a combination of elderberry and reishi extracts for 14 weeks^40^—compared with placebo. Neither found a significant effect on incidence of respiratory infections (OR 1.24, 95%CI 0.76–2.01; IRR 1.06, 95%CI 0.51–2.18 respectively). Mild adverse effects were reported with the Chinese formula, but these only lasted 2–3 days (OR 1.41, 95%CI 1.02–1.96). There was no significant increase in adverse events with the elderberry and reishi extracts.

## Discussion

### Main findings of this study

Overall, we found limited evidence for interventions to reduce transmission of ARIs in care homes. Interventions to improve hand hygiene and infection control had mixed results in four cluster RCTs, with only one study showing a reduction in pneumonia incidence, one showing a reduction in influenza outbreaks, and one showing a reduction in incidence of ILI. Achieving high levels of adherence to infection control is challenging, and interventions were all different.

Antiviral prophylaxis at the time of an outbreak gave promising results. Oseltamivir seemed to halve the risk of SCLI and ILI, and to have a dose–response effect. A longer course had a larger effect, and the effect on SCLI was greater than the effect on all laboratory-confirmed influenza (perhaps viral load was reduced rather than being completely eliminated). A single dose of bamlanivimab was effective in preventing COVID-19 infections after an index case, in an unvaccinated population at the start of the pandemic. However, some of these studies were at high ROB so results should be interpreted with caution. Although there were 10 RCTs of nutritional supplements, only high-dose Vitamin D3 led to a significant reduction in ARIs, in a single trial.

### What is already known on this topic

Face masks have now become a standard part of infection control, but all the ‘hygiene’ trials in this review predate the COVID-19 pandemic so did not include them in their interventions. Current evidence for effectiveness of face masks is mixed,^42^^,^^43^ and would warrant further investigation in well-designed, pragmatic studies.

Deaths in influenza outbreaks are significantly more frequent in homes which do not use oseltamivir prophylaxis.^44^ However, in 2008–9, >90% of influenza A viruses tested had become resistant to oseltamivir.^45^^,^^46^ Although these were replaced by sensitive strains in the 2009 pandemic,^47^ resistant strains will probably re-emerge. Severe acute respiratory syndrome coronavirus 2 (SARS-CoV-2) also rapidly developed resistance to bamlanivimab.^48–50^ Although initial results were promising, the Food and Drug Administration subsequently revoked its use as a monotherapy.^51^ Furthermore, the RCT of bamlanivimab was in a population unvaccinated for SARS-CoV-2, so its prophylactic efficacy in the current vaccinated population is unknown.

Unlike the trial of high-dose vitamin D3, which appeared to show an increase risk of falls, a large meta-analysis has shown that vitamin D3 supplementation reduces the risk of falls in the elderly.^52^ Other studies have suggested that Vitamin D3 may have other benefits including prevention of COVID-19^53^ and improved cognitive function.^54^

### What this study adds

Infection control is the key to reduce transmission of respiratory infections. However, current evidence is insufficient to recommend any particular intervention to improve its implementation by care home staff.

Current NICE guidelines recommend antiviral prophylaxis with oseltamivir or zanamivir for care home residents during localized outbreaks of influenza,^55^ but often this guidance is not implemented.^56^ All the studies in this review confirm a significant benefit in care home residents, even those who had been vaccinated. Better systems are needed to implement prophylaxis, which requires testing care home residents with ILI for influenza virus, and evaluating their renal function, as oseltamivir is contraindicated if the estimated Glomerular Filtration Rate (eGFR) is < 10 ml/min/1.73 m^2^ (all trials excluded participants with significant renal impairment).

The simplest, most promising intervention is supplementation with high-dose Vitamin D3, which reduced incidence of ARIs by 40%.^32^ Based on current evidence, other supplements cannot be recommended for preventing respiratory infections in care home residents.

Further research is needed to improve evidence on all promising interventions, given the major impact of ARI outbreaks on care home residents. It would be especially useful to repeat the high-dose vitamin D3 trial in a larger sample, to check whether the same result can be replicated. If so, routine supplementation with high-dose vitamin D3 could be a cost-effective strategy for reducing risk of ARIs in care homes.

More impactful behaviour-change interventions are needed to improve infection control, designed using behavioural science and the person-based approach^57^ and including not only hand washing but also other non-pharmacological measures. However, the impact of such interventions will always be limited because they are aimed at staff, whereas much of the transmission of infections is likely to be between residents.

Antiviral prophylaxis is a promising strategy as long as the virus remains sensitive and incident cases of influenza or COVID-19 are detected early. Further trials are needed to evaluate whether drug combinations are well-tolerated, effective, and could prevent emergence of resistant strains.

It is also important to evaluate the effectiveness of vaccines, which will be the subject of a subsequent systematic review.

### Limitations

We conducted a comprehensive search of the literature, with no exclusions based on language or year of publication. The main limitation is that we did not search the grey literature.

## Conclusion

Transmission of respiratory infections in care homes can be reduced by certain educational interventions to improve implementation of infection control by staff, by antiviral prophylaxis soon after a case of influenza or COVID-19 has been detected, and by continuous routine supplementation with high-dose Vitamin D3. Other routine supplements did not reduce the risk of respiratory infections.

## Supplementary Material

Supplementary_SearchStrategies_fdae178

## Data Availability

All data are from published studies which are referenced below. We did not generate any new data for this systematic review.

## Notes

**Merlin L. Willcox,** Principal Research Fellow

**Deepthi Lavu,** GP Academic Clinical Fellow

**Usaid Yousaf,** Medical Student

**Sam Dalton,** Medical Student

**Nia Roberts,** Senior Outreach Librarian

**Annette Plüddemann,** Senior Research Fellow
