# Supplementary material for: The effectiveness of interventions to reduce the transmission of acute respiratory infections in care homes: a systematic review
Source: J Public Health (Oxf). 2024 Aug 13;46(4):551–63. doi: 10.1093/pubmed/fdae178 (PMC11637680; doi:10.1093/pubmed/fdae178)
Supplement: Supplementary_SearchStrategies_fdae178 [file supplementary_searchstrategies_fdae178.docx]

Supplementary Materials: Full Search Strategies

# Medline

| 1 | exp nursing homes/ or residential facilities/ or assisted living facilities/ or halfway houses/ or "homes for the aged"/ or group homes/ | 57562 |
| --- | --- | --- |
| 2 | hospitals, veterans/ | 6915 |
| 3 | long term care/ | 28315 |
| 4 | ((care or nursing or healthcare or residential) adj2 (facility or facilities or home?)).ti,ab. | 106514 |
| 5 | (group home? or halfway hous* or half-way hous* or halfway home? or half-way home? or assisted living).ti,ab. | 3930 |
| 6 | ((care or convalescent) adj (home? or center? or centre? or facility or facilities)).ti,ab. | 71128 |
| 7 | ((skilled or intermediate) adj (nursing facility or nursing facilities)).ti,ab. | 3473 |
| 8 | (resident* adj2 care).ti,ab. | 10578 |
| 9 | ((longterm or long term) adj3 (care or facility or facilities)).ti,ab. | 30371 |
| 10 | or/1-9 | 209756 |
| 11 | respiratory tract infections/ or exp bronchitis/ or common cold/ or exp empyema, pleural/ or influenza, human/ or laryngitis/ or exp pharyngitis/ or exp pleurisy/ or exp pneumonia/ or rhinitis/ or exp sinusitis/ or exp supraglottitis/ or tracheitis/ or Cough/ or Sneezing/ or exp Otitis Media/ or Earache/ | 529540 |
| 12 | (ARI or ARTI or URTI or LRTI or LRI).ti,ab. | 7896 |
| 13 | exp COVID-19/ | 218713 |
| 14 | (pharyngit* or nasopharyngit* or naso-pharyngit* or rhinopharyngit* or rhino-pharyngit* or sinusit* or nasosinusit* or naso-sinusit* or rhinosinusit* or rhino-sinosit* or rhinit* or rhinorrhoea or rhinorrhea or ((runny or running or discharg* or congest* or blocked or stuff or dripping) adj2 nose?)).ti,ab. | 66421 |
| 15 | (common cold or throat infection or sore throat or throat pain or inflammed throat or tonsilitis or cough or sneez* or otitis media or earache).ti,ab. | 90999 |
| 16 | (2019 nCoV or 2019nCoV or corona virus* or coronavirus* or COVID* or nCov 2019 or SARS-CoV2 or SARS CoV-2 or SARSCoV2 or SARSCoV-2).ti,ab. | 353232 |
| 17 | (bronchitis or bronchiolitis or chest infection? or pneumonia? or pleurisy).ti,ab. | 236917 |
| 18 | or/11-17 | 876224 |
| 19 | 10 and 18 | 14378 |
| 20 | randomized controlled trial.pt. | 590620 |
| 21 | controlled clinical trial.pt. | 95262 |
| 22 | randomized.ab. | 599328 |
| 23 | placebo.ab. | 237335 |
| 24 | clinical trials as topic.sh. | 200936 |
| 25 | randomly.ab. | 405974 |
| 26 | trial.ti. | 282992 |
| 27 | 20 or 21 or 22 or 23 or 24 or 25 or 26 | 1516018 |
| 28 | exp animals/ not humans.sh. | 5111074 |
| 29 | 27 not 28 | 1395123 |
| 30 | 19 and 29 | 829 |

# Embase (1974 to 23 April 2023)

| 1 | assisted living facility/ or nursing home/ or residential home/ or halfway house/ | 71636 |
| --- | --- | --- |
| 2 | long term care/ | 145211 |
| 3 | ((care or nursing or healthcare or residential) adj2 (facility or facilities or home?)).ti,ab. | 139694 |
| 4 | (group home? or halfway hous* or half-way hous* or halfway home? or half-way home? or assisted living).ti,ab. | 5182 |
| 5 | ((care or convalescent) adj (home? or center? or centre? or facility or facilities)).ti,ab. | 108985 |
| 6 | ((skilled or intermediate) adj (nursing facility or nursing facilities)).ti,ab. | 6058 |
| 7 | (resident* adj2 care).ti,ab. | 14026 |
| 8 | ((longterm or long term) adj3 (care or facility or facilities)).ti,ab. | 40004 |
| 9 | or/1-8 | 379034 |
| 10 | respiratory infection/ or nose infection/ or upper respiratory tract infection/ or common cold/ or rhinopharyngitis/ or lower respiratory tract infection/ or chest infection/ or lung infection/ or exp otitis media/ or exp bronchitis/ or pleurisy/ or pleura empyema/ or exp coughing/ or sneezing/ or respiratory tract inflammation/ or exp laryngitis/ or exp pharyngitis/ or exp rhinitis/ or exp sinusitis/ or exp tonsillitis/ or exp tracheitis/ | 537151 |
| 11 | (ARI or ARTI or URTI or LRTI or LRI).ti,ab. | 11541 |
| 12 | coronavirus disease 2019/ | 344214 |
| 13 | (pharyngit* or nasopharyngit* or naso-pharyngit* or rhinopharyngit* or rhino-pharyngit* or sinusit* or nasosinusit* or naso-sinusit* or rhinosinusit* or rhino-sinosit* or rhinit* or rhinorrhoea or rhinorrhea or ((runny or running or discharg* or congest* or blocked or stuff or dripping) adj2 nose?)).ti,ab. | 95921 |
| 14 | (common cold or throat infection or sore throat or throat pain or inflammed throat or tonsilitis or cough or sneez* or otitis media or earache).ti,ab. | 140276 |
| 15 | (2019 nCoV or 2019nCoV or corona virus* or coronavirus* or COVID* or nCov 2019 or SARS-CoV2 or SARS CoV-2 or SARSCoV2 or SARSCoV-2).ti,ab. | 432905 |
| 16 | (bronchitis or bronchiolitis or chest infection? or pneumonia? or pleurisy).ti,ab. | 336467 |
| 17 | or/10-16 | 1247131 |
| 18 | 9 and 17 | 27541 |
| 19 | randomized controlled trial/ | 779190 |
| 20 | double blind procedure/ or single blind procedure/ | 258265 |
| 21 | crossover procedure/ | 74834 |
| 22 | random*.tw. | 1951957 |
| 23 | (((singl* or doubl*) adj (blind* or mask*)) or crossover or cross over or factorial* or latin square or assign* or allocat* or volunteer*).ti,ab. | 1284919 |
| 24 | randomly.ab. | 546119 |
| 25 | trial.ti. | 399293 |
| 26 | 19 or 20 or 21 or 22 or 23 | 2817501 |
| 27 | (exp animals/ or nonhuman/) not human/ | 7277060 |
| 28 | 26 not 27 | 2475303 |
| 29 | 18 and 28 | 3285 |

# CINAHL

| **#** | **Query** | **Results** |
| --- | --- | --- |
| S47 | S23 AND S46 | 1,005 |
| S46 | S39 not S45 | 956,811 |
| S45 | S44 not S43 | 212,664 |
| S44 | S40 OR S41 OR S42 | 246,409 |
| S43 | MH (human) | 2,659,728 |
| S42 | TI (animal model*) | 3,685 |
| S41 | MH (animal studies) | 151,565 |
| S40 | MH animals+ | 103,743 |
| S39 | S24 OR S25 OR S26 OR S27 OR S28 OR S29 OR S30 OR S31 OR S32 OR S33 OR S34 OR S35 OR S36 OR S37 OR S38 | 1,003,977 |
| S38 | AB (cluster W3 RCT) | 488 |
| S37 | MH (crossover design) OR MH (comparative studies) | 471,505 |
| S36 | AB (control W5 group) | 142,994 |
| S35 | PT (randomized controlled trial) | 150,038 |
| S34 | MH (placebos) | 13,675 |
| S33 | MH (sample size) AND AB (assigned OR allocated OR control) | 4,450 |
| S32 | TI (trial) | 178,145 |
| S31 | AB (random*) | 395,601 |
| S30 | TI (randomised OR randomized) | 138,533 |
| S29 | MH cluster sample | 5,225 |
| S28 | MH pretest-posttest design | 52,375 |
| S27 | MH random assignment | 78,465 |
| S26 | (MH single-blind studies) | 15,956 |
| S25 | (MH double-blind studies) | 54,027 |
| S24 | (MH randomized controlled trials) | 136,844 |
| S23 | S14 AND S22 | 7,378 |
| S22 | S15 OR S16 OR S17 OR S18 OR S19 OR S20 OR S21 | 245,540 |
| S21 | ((TI Influenza OR AB Influenza) OR (TI Flu OR AB Flu) OR (TI H1N1 OR AB H1N1) OR ((TI nCoV OR AB nCoV) OR (TI 2019nCoV OR AB 2019nCoV) OR (TI "corona virus*" OR AB "corona virus*") OR (TI coronavirus* OR AB coronavirus*) OR (TI COVID* OR AB COVID*) OR (TI "nCov 2019" OR AB "nCov 2019") OR (TI SARS-CoV2 OR AB SARS-CoV2) OR (TI "SARS CoV-2" OR AB "SARS CoV-2") OR (TI SARSCoV2 OR AB SARSCoV2) OR (TI SARSCoV-2 OR AB SARSCoV-2))) | 149,008 |
| S20 | (TI bronchitis or TI bronchiolitis or TI "chest infection" or TI pneumonia* or TI pleurisy OR AB bronchitis or AB bronchiolitis or AB "chest infection" or AB pneumonia* or AB pleurisy) | 45,086 |
| S19 | ((TI "throat infection" OR AB "throat infection") OR (TI "sore throat" OR AB "sore throat") OR (TI "throat pain" OR AB "throat pain") OR (TI "inflammed throat" OR AB "inflammed throat") OR (TI tonsillitis OR AB tonsillitis) OR (TI cough OR AB cough) OR (TI sneez* OR AB sneez*) OR (TI "common cold" or AB "common cold") or (TI "otitis media" or AB "otitis media" or TI earache OR AB earache)) | 23,052 |
| S18 | ((TI pharyngit* OR AB pharyngit*) OR (TI nasopharyngit* OR AB nasopharyngit*) OR (TI naso-pharyngit* OR AB naso-pharyngit*) OR (TI rhinopharyngit* OR AB rhinopharyngit*) OR (TI rhino-pharyngit* OR AB rhino-pharyngit*) OR (TI sinusit* OR AB sinusit*) OR (TI nasosinusit* OR AB nasosinusit*) OR (TI naso-sinusit* OR AB naso-sinusit*) OR (TI rhinosinusit* OR AB rhinosinusit*) OR (TI rhino-sinosit* OR AB rhino-sinosit*) OR (TI rhinit* OR AB rhinit*) OR (TI rhinorrhoea OR AB rhinorrhoea) OR (TI rhinorrhea OR AB rhinorrhea) OR (((TI runny OR AB runny) OR (TI running OR AB running) OR (TI discharg* OR AB discharg*) OR (TI congest* OR AB congest*) OR (TI blocked OR AB blocked) OR (TI stuff OR AB stuff) OR (TI dripping OR AB dripping)) N2 (TI nose# OR AB nose#))) | 12,470 |
| S17 | (MH COVID-19+) | 40,634 |
| S16 | ((TI ARI OR AB ARI) OR (TI ARTI OR AB ARTI) OR (TI URTI OR AB URTI) OR (TI LRTI OR AB LRTI) OR (TI LRI OR AB LRI)) | 3,162 |
| S15 | (MH "respiratory tract infections") OR (MH bronchitis+) OR (MH "common cold") OR (MH "empyema, pleural"+) OR (MH "influenza, human") OR (MH laryngitis) OR (MH pharyngitis+) OR (MH pleurisy+) OR (MH pneumonia+) OR (MH rhinitis) OR (MH sinusitis+) OR (MH supraglottitis+) OR (MH tracheitis) OR (MH Cough) OR (MH Sneezing) OR (MH "Otitis Media"+) OR (MH Earache) | 70,807 |
| S14 | S1 OR S2 OR S3 OR S4 OR S5 OR S6 OR S7 OR S8 OR S9 OR S10 OR S11 OR S12 OR S13 | 148,894 |
| S13 | (TI "halfway hous*" or AB "halfway hous* or TI "half-way hous*" or AB "half-way hous* or TI "halfway home*" or AB "halfway home*" or TI "half-way home*" or AB "half-way home*") | 33 |
| S12 | (MH "halfway houses") | 214 |
| S11 | (MH "residential facilities") | 5,362 |
| S10 | ((TI healthcare OR AB healthcare) N2 ((TI facility OR AB facility) OR (TI facilities OR AB facilities))) | 9,169 |
| S9 | (((TI longterm OR AB longterm) OR (TI "long term" OR AB "long term")) N3 ((TI care OR AB care) OR (TI facility OR AB facility) OR (TI facilities OR AB facilities))) | 23,707 |
| S8 | (MH "long term care") | 28,107 |
| S7 | ((TI resident* OR AB resident*) N2 (TI care OR AB care OR TI facility OR AB facility OR TI facilities OR AB facilities)) | 13,243 |
| S6 | (((TI skilled OR AB skilled) OR (TI intermediate OR AB intermediate)) W1 ((TI "nursing facility" OR AB "nursing facility") OR (TI "nursing facilities" OR AB "nursing facilities"))) | 2,900 |
| S5 | (((TI care OR AB care) OR (TI convalescent OR AB convalescent)) W1 ((TI home# OR AB home#) OR (TI center# OR AB center#) OR (TI centre# OR AB centre#) OR (TI facility OR AB facility) OR (TI facilities OR AB facilities))) | 43,806 |
| S4 | (MH "hospitals, veterans") | 5,259 |
| S3 | (((TI care OR AB care) OR (TI nursing OR AB nursing) OR (TI healthcare OR AB healthcare) OR (TI residential OR AB residential)) N2 ((TI facility OR AB facility) OR (TI facilities OR AB facilities) OR (TI home# OR AB home#))) | 82,168 |
| S2 | (MH "nursing homes") | 25,828 |
| S1 | (TI Group home# or AB group home# or TI "assisted living" or AB "assisted living") | 8,247 |

# Cochrane Central

| ID | Search | Hits |
| --- | --- | --- |
| #1 | (((care OR nursing OR healthcare OR residential) NEAR/2 (facility OR facilities OR home*))):ti,ab,kw | 17140 |
| #2 | (((group NEXT home*) or ((halfway or half-way) NEXT (hous* or home*)) or (assisted NEXT living))):ti,ab,kw | 666 |
| #3 | (((care OR convalescent) NEXT (home* OR center* OR centre* OR facility OR facilities))):ti,ab,kw | 12840 |
| #4 | (((skilled OR intermediate) NEXT ("nursing facility" OR "nursing facilities"))):ti,ab,kw | 312 |
| #5 | ((resident* NEAR/2 (care OR facility OR facilities))):ti,ab,kw | 1947 |
| #6 | (((longterm OR "long term") NEAR/3 (care OR facility OR facilities))):ti,ab,kw | 8544 |
| #7 | MeSH descriptor: [Long-Term Care] explode all trees | 1351 |
| #8 | MeSH descriptor: [Residential Facilities] explode all trees | 2300 |
| #9 | #1 OR #2 OR #3 OR #4 OR #5 OR #6 OR #7 OR #8 | 32641 |
| #10 | MeSH descriptor: [Respiratory Tract Infections] this term only | 2771 |
| #11 | MeSH descriptor: [Bronchitis] explode all trees | 2298 |
| #12 | MeSH descriptor: [Common Cold] explode all trees | 663 |
| #13 | MeSH descriptor: [Empyema, Pleural] explode all trees | 69 |
| #14 | MeSH descriptor: [Influenza, Human] explode all trees | 3224 |
| #15 | MeSH descriptor: [Laryngitis] explode all trees | 159 |
| #16 | MeSH descriptor: [Pharyngitis] explode all trees | 1601 |
| #17 | MeSH descriptor: [Pleurisy] explode all trees | 53 |
| #18 | MeSH descriptor: [Pneumonia] explode all trees | 9755 |
| #19 | MeSH descriptor: [Rhinitis] explode all trees | 4629 |
| #20 | MeSH descriptor: [Sinusitis] explode all trees | 1393 |
| #21 | MeSH descriptor: [Supraglottitis] explode all trees | 11 |
| #22 | MeSH descriptor: [Tracheitis] explode all trees | 32 |
| #23 | MeSH descriptor: [Cough] explode all trees | 1609 |
| #24 | MeSH descriptor: [Sneezing] explode all trees | 268 |
| #25 | MeSH descriptor: [Otitis Media] explode all trees | 1390 |
| #26 | MeSH descriptor: [Earache] explode all trees | 42 |
| #27 | ((ARI OR ARTI OR URTI OR LRTI OR LRI)):ti,ab,kw | 1613 |
| #28 | MeSH descriptor: [COVID-19] explode all trees | 4093 |
| #29 | ((pharyngit* OR nasopharyngit* OR naso-pharyngit* OR rhinopharyngit* OR rhino-pharyngit* OR sinusit* OR nasosinusit* OR naso-sinusit* OR rhinosinusit* OR rhino-sinosit* OR rhinit* OR rhinorrhoea OR rhinorrhea OR ((runny OR running OR discharg* OR congest* OR blocked OR stuff OR dripping) NEAR/2 nose))):ti,ab,kw | 21006 |
| #30 | (("common cold" or "throat infection" OR "sore throat" OR "throat pain" OR "inflammed throat" OR tonsilitis OR cough OR sneez* or "otitis media" or earache)):ti,ab,kw | 20705 |
| #31 | ((Influenza OR "Flu outbreak" OR H1N1 OR (nCoV OR 2019nCoV OR ("corona" NEXT virus*) OR coronavirus* OR COVID* OR "nCov 2019" OR SARS-CoV2 OR "SARS CoV-2" OR SARSCoV2 OR SARSCoV-2))):ti,ab,kw | 24494 |
| #32 | ((bronchitis or bronchiolitis or "chest infection*" or pneumonia* or pleurisy)):ti,ab,kw | 27598 |
| #33 | #10 or #11 or #12 or #13 or #14 or #15 or #16 or #17 or #18 or #19 or #20 or #21 or #22 or #23 or #24 or #25 or #26 or #27 or #28 or #29 or #30 or #31 or #32 | 83251 |
| #34 | #9 and #33 in Trials | 2146 |
| #35 | #9 and #33 in Cochrane Reviews | 35 |

# Europe PMC

| (TITLE:"nursing home" OR TITLE:"nursing homes" OR TITLE:"care home" OR TITLE:"care homes" OR TITLE:"residential home" OR TITLE:"residential homes") AND (TITLE:respiratory OR TITLE:covid OR TITLE:coronavirus OR TITLE:"common cold" OR Title:pneumonia OR TITLE:bronchitis OR TITLE:pharyngitis OR TITLE:nasopharyngitis OR TITLE:naso-pharyngitis OR TITLE:rhinits OR TITLE:rhinopharygtitis OR TITLE:rhino-pharyngitis OR TITLE:sinusitis OR TITLE:nasosinusitus OR TITLE:naso-sinusitis OR TITLE:pleurisy OR TITLE:empyema OR TITLE:cough OR TITLE:tonsillitis OR TITLE:sneeze OR TITLE:sneezing) AND (SRC:"PPR") | 128 |
| --- | --- |
| (ABSTRACT:"nursing home" OR ABSTRACT:"nursing homes" OR ABSTRACT:"care home" OR ABSTRACT:"care homes" OR ABSTRACT:"residential home" OR ABSTRACT:"residential homes") AND (ABSTRACT:respiratory OR ABSTRACT:covid OR ABSTRACT:coronavirus OR ABSTRACT:"common cold" OR ABSTRACT:pneumonia OR ABSTRACT:bronchitis OR ABSTRACT:pharyngitis OR ABSTRACT:nasopharyngitis OR ABSTRACT:naso-pharyngitis OR ABSTRACT:rhinits OR ABSTRACT:rhinopharygtitis OR ABSTRACT:rhino-pharyngitis OR ABSTRACT:sinusitis OR ABSTRACT:nasosinusitus OR ABSTRACT:naso-sinusitis OR ABSTRACT:pleurisy OR ABSTRACT:empyema OR ABSTRACT:cough OR ABSTRACT:tonsillitis OR ABSTRACT:sneeze OR ABSTRACT:sneezing) AND (SRC:"PPR") | 420 |
|  | 548 |

# ClinicalTrials.gov

| Other terms=("nursing home" OR "nursing homes" OR "care home" OR "care homes" OR "residential home" OR "residential homes") AND Condition=(respiratory OR covid OR coronavirus OR "common cold" OR pneumonia OR bronchitis OR pharyngitis OR nasopharyngitis OR naso-pharyngitis OR rhinits OR rhinopharygtitis OR rhino-pharyngitis OR sinusitis OR nasosinusitus OR naso-sinusitis OR pleurisy) | 188 |
| --- | --- |
| Other terms=("nursing home" OR "nursing homes" OR "care home" OR "care homes" OR "residential home" OR "residential homes") AND Condition=(empyema OR cough OR tonsilitis OR sneeze OR sneezing) | 2 |
| Title=("nursing home" OR "nursing homes" OR "care home" OR "care homes" OR "residential home" OR "residential homes") AND Condition=(empyema OR cough OR tonsilitis OR sneeze OR sneezing) | 0 |
| Title=("nursing home" OR "nursing homes" OR "care home" OR "care homes" OR "residential home" OR "residential homes") AND Condition=(respiratory OR covid OR coronavirus OR "common cold" OR pneumonia OR bronchitis OR pharyngitis OR nasopharyngitis OR naso-pharyngitis OR rhinits OR rhinopharygtitis OR rhino-pharyngitis OR sinusitis OR nasosinusitus OR naso-sinusitis OR pleurisy) | 74 |
|  | 264 |
